# Increased frequency of circulating Th22 cells in patients with B-cell non-Hodgkin's lymphoma

**DOI:** 10.18632/oncotarget.10966

**Published:** 2016-07-30

**Authors:** Ting Lu, Yan Liu, Shuang Yu, Congcong Yin, Peng Li, Jingjing Ye, Daoxin Ma, Chunyan Ji

**Affiliations:** ^1^ Department of Hematology, Qilu Hospital, Shandong University, Jinan, PR China

**Keywords:** B-cell non-Hodgkin's lymphoma, Th22, IL-22, prognosis

## Abstract

T helper (Th) 22 cells play important roles in the pathogenesis of autoimmune and inflammatory diseases, and their function in tumors remains uncertain. In the current study, we investigated the alternations and clinical significance of circulating Th22 cells in patients with B-cell non-Hodgkin's lymphoma (B-NHL). We found that the frequency of Th22 cells was significantly elevated in peripheral blood of newly-diagnosed B-NHL patients, and returned to normal level after chemotherapy. In consistent with increased Th22 frequency, plasma IL-22 and IL-6 levels in B-NHL patients were remarkably increased. Moreover, the increased Th22 frequency was associated with the older age (> 60 yr) and a poorer response to therapy in B-NHL patients. In addition, there existed a statistically positive correlation between circulating Th22 and Th17 frequencies in B-NHL patients. Our data demonstrated that circulating Th22 frequency was associated with the clinical outcome and prognosis of B-NHL patients, indicating that Th22 immune response might play an important role in the development and progression of B-NHL.

## INTRODUCTION

Non-Hodgkin's lymphoma (NHL) is a heterogeneous group of malignancies originating in lymphatic hematopoietic tissue. According to the type of lymphoid cell, NHL is classified into B-cell lymphoma and T-cell lymphoma. The pathogenesis and development of lymphoma is complicated, and the detail mechanisms remain unclear.

Abundant evidences have demonstrated that T helper (Th) cells including Th1, Th2 and Th17 cells participate in the development and progression of different tumors [1–4]. Th22 cells are a newly identified Th subset, which is characterized by secretion of IL-22 and TNF-α, but not IFN-γ (Th1 marker) or IL-17 (Th17 marker) [5, 6]. The naive CD4^+^ T cells can differentiate toward the Th22 phenotype in the presence of IL-6 and tumor necrosis factors (TNF)-α with the aid of plasmacytoid dendritic cells [6]. IL-22 is the main effector cytokine of Th22 subset, which belongs to the IL-10 cytokine family [7, 8]. IL-22 binds to a heterodimeric receptor complex that consists of IL-22 receptor (R) 1 and IL-10R2. Therefore, IL-22 functions as a signaling mediator between the immune system and non-hematological environment [9–11]. And it can activate a number of intracellular signaling pathways including JAK-STAT3 and MAPK pathway [12–14].

Th22 cells have been revealed to play critical roles in the pathogenesis of inflammatory and autoimmunity diseases [15–18]. Based on the present understandings of IL-22 biology, it is believed that IL-22 is important in enhancing innate immunity response, tissue damage protection and tissue repair [19]. However, the role of Th22 cells in tumor immunity remains uncertain. A few reports showed that Th22 cells were associated with the progression and development in hepatocellular carcinoma [20], gastric cancer [21, 22] and cervical cancer [23]. Up to now, no previous study has shown evidence regarding the role of Th22 subset and its related cytokines in the progression and development of B-NHL.

Therefore, in order to identify and describe the possible roles of Th22 in B-NHL, we investigated the frequency of Th22 (CD4^+^IFN-γ^−^IL-17^−^IL-22^+^) cells in the peripheral blood, and the concentrations of plasma IL-22, IL-6 and TNF-α in patients with B-NHL. We also evaluated the relevance between Th22 frequency and clinical characteristics or disease's development in this study.

## RESULTS

### Circulating Th22 frequency was elevated in newly-diagnosed non-Hodgkin's lymphoma rather than Hodgkin's lymphoma

**Figure 1:**
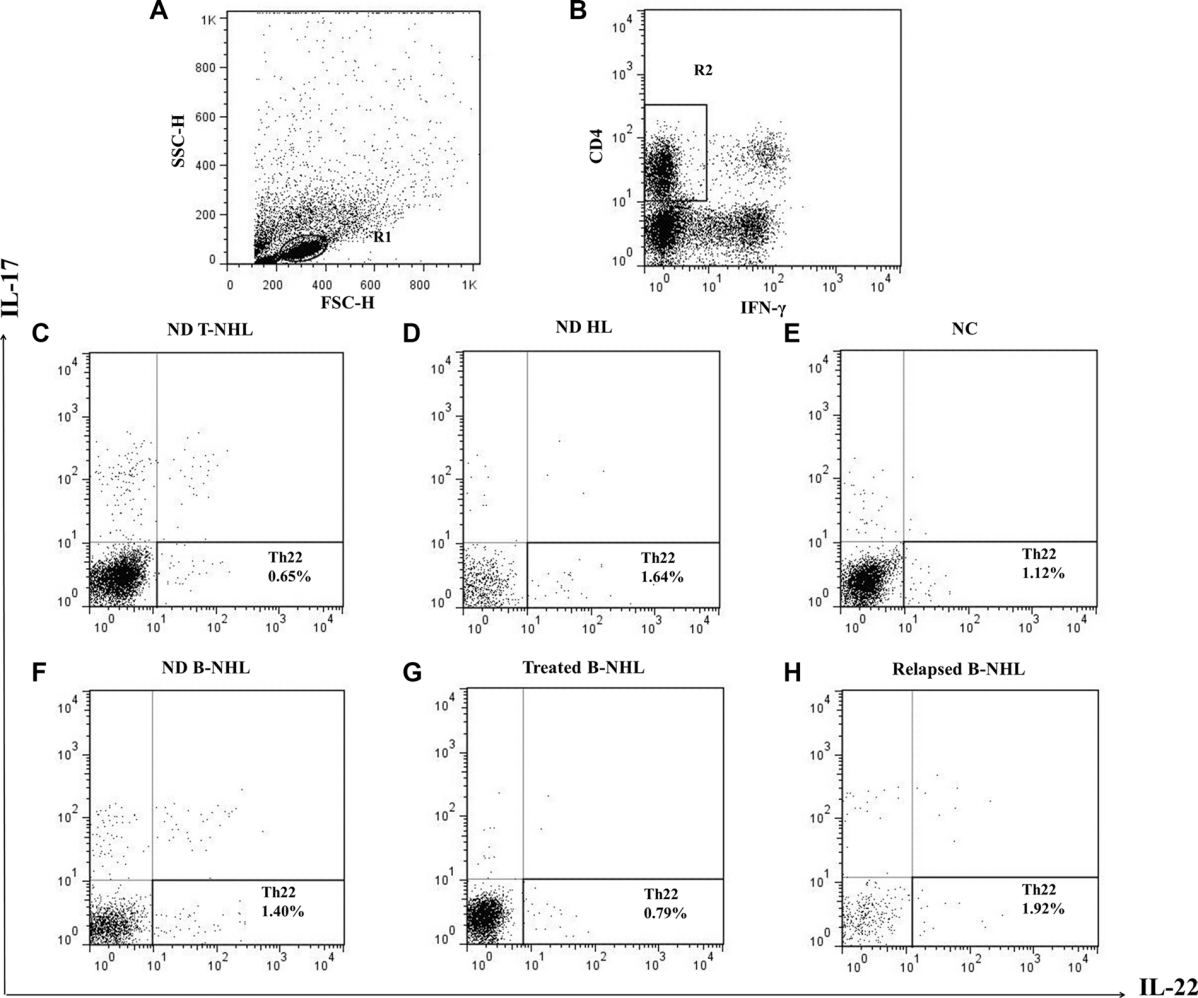
Circulating frequencies of Th22 cells in representative patients and normal controls (**A**) Lymphocytes were gated in R1. (**B**) CD4^+^IFN-γ^−^ T cells were gated in R2. (**C**–**F**) Representative FACS dot plots of Th22 (CD4^+^IFN-γ^−^IL-17^−^IL-22^+^) cells as a proportion of CD4^+^IFN-γ^−^ T cells from newly-diagnosed (ND) B-NHL, T-NHL, HL patients and normal controls (NC). (**G**) Representative FACS dot plots of Th22 cells in treated B-NHL patients. (**H**) Representative FACS dot plots of Th22 cells in relapsed B-NHL patients.

**Figure 2:**
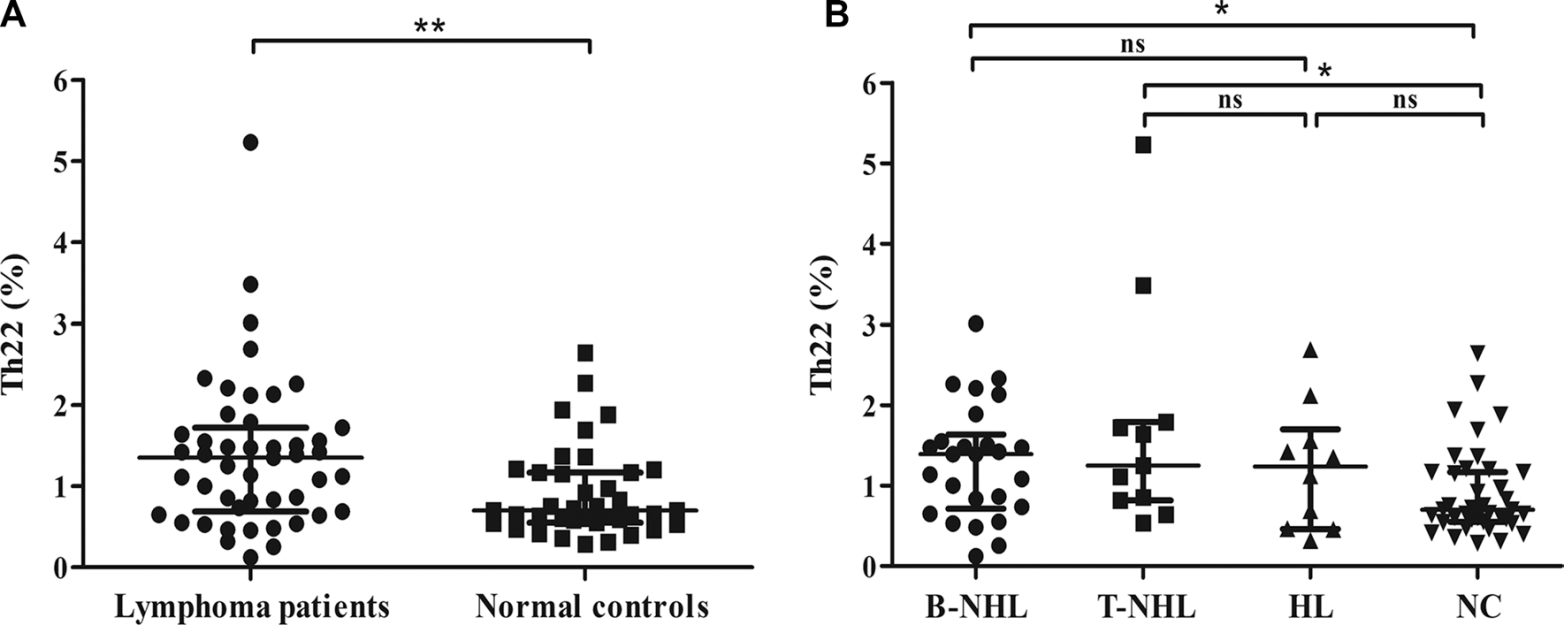
Results of circulating Th22 cells (**A**) The frequency of circulating Th22 cells was significantly higher in lymphoma patients than normal controls. (**B**) Circulating Th22 frequencies were remarkably increased in B-NHL patients and T-NHL patients rather than HL patients compared with those in controls. *P < 0.05; **P < 0.01; ns, non-significant.

The typical dot plots of Th22 cells in representative lymphoma patients and normal controls were shown in Figure 1A–1F. Compared with normal controls (median, 0.656% of CD4^+^IFN-γ^−^ T cells; range, 0.286–2.640%; *n* = 39), the frequency of circulating Th22 cells was significantly elevated in newly-diagnosed patients with lymphoma (1.350%; 0.124–5.230%; *n* = 47; *P* = 0.0053) (Figure 2A). Furthermore, those lymphoma patients were divided into three groups, B-cell non-Hodgkin's lymphoma (B-NHL), T-cell non-Hodgkin's lymphoma (T-NHL) and Hodgkin's lymphoma (HL). A remarkable increase of Th22 frequency was found in B-NHL (1.390%; 0.124–3.01%; *n* = 26; *P* = 0.0147) and T-NHL (1.250%; 0.537–5.230%; *n* = 11; *P* = 0.0204) rather than in HL (1.235%; 0.321–2.69%; *n* = 10; *P* = 0.1464), compared with those in controls (Figure 2B). Meanwhile, there was no significant difference of circulating Th22 frequencies between B-NHL and T-NHL with HL group (*P* = 0.7727; *P* = 0.3224). Additionally, no significant difference of Th22 distribution was found in subtypes of B-NHL and T-NHL (Supplementary Figure S1A and S1B).

### Plasma IL-22 level was up-regulated in newly-diagnosed B-NHL patients, accompanied with elevated IL-6 and normal TNF-α

**Figure 3:**
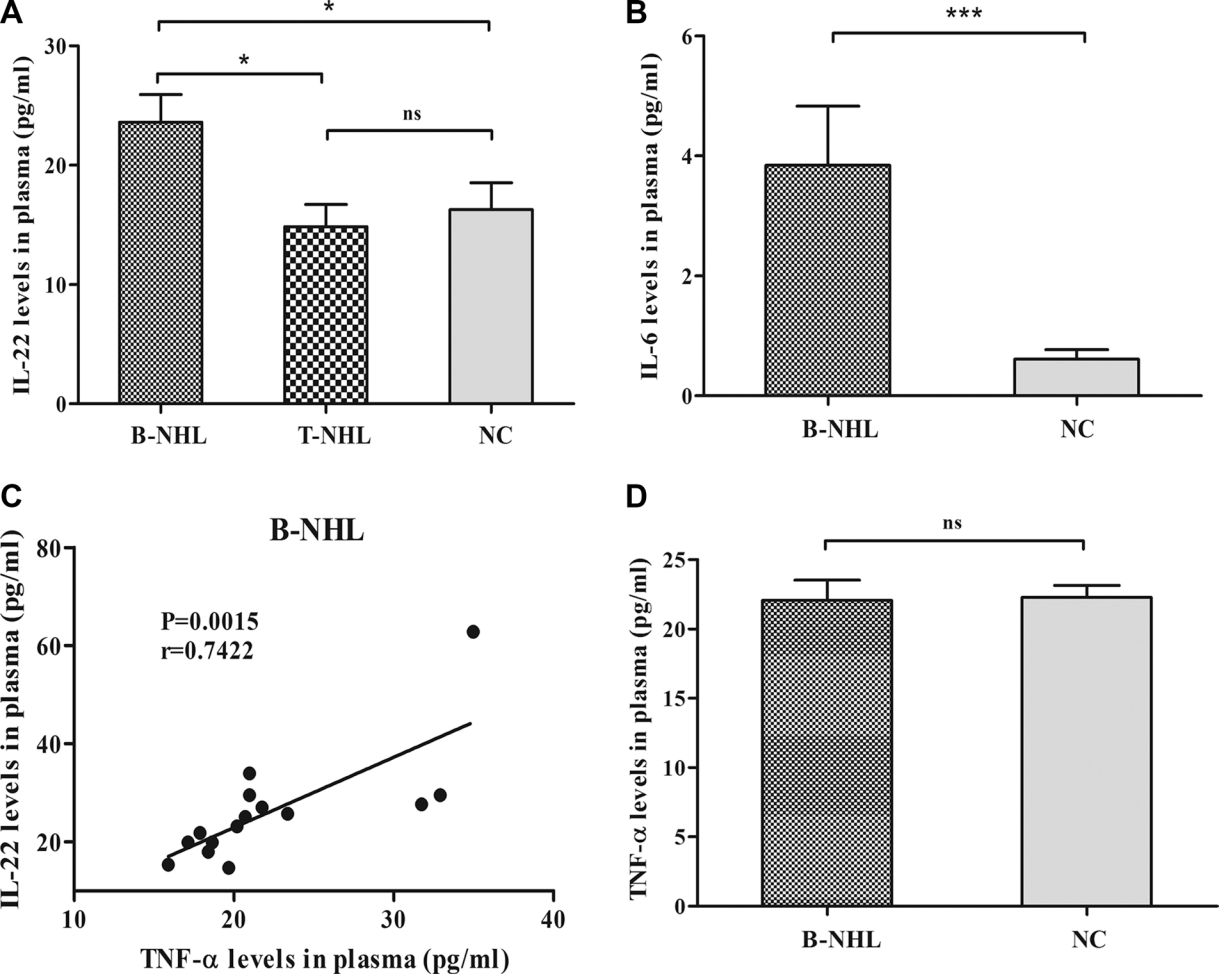
Concentrations of Th22 cells related cytokines (**A**) Concentration of plasma IL-22 in B-NHL patients was significantly elevated than that in T-NHL patients and healthy controls. (**B**) Concentration of plasma IL-6 in B-NHL patients was significantly increased than that in healthy controls. (**C**) A positive correlation was found between plasma IL-22 and TNF-α in B-NHL. (**D**) Concentration of plasma TNF-α level in B-NHL patients was similar to that in normal individuals. *P < 0.05; ***P < 0.001; ns, non-significant.

The levels of IL-22, IL-6 and TNF-α in peripheral blood were measured by ELISA. Compared with normal controls (5 samples undetectable; 16.27 ± 2.245 pg/ml; *n* = 29), plasma IL-22 level was significantly increased in newly-diagnosed B-NHL patients (23.61 ± 2.304 pg/ml; *n* = 22; *P* = 0.0292) rather than in T-NHL patients (14.84 ± 1.859 pg/ml; *n* = 8; *P* = 0.7961) (Figure 3A). And IL-22 concentration in B-NHL was even higher than that in T-NHL (*P* = 0.0374) (Figure 3A). Meanwhile, we detected plasma IL-6 concentration and found a dramatic increase in B-NHL patients (1 sample undetectable; undetectable to 13.55 pg/ml; *n* = 15) compared with normal controls (16 samples undetectable; undetectable to 2.65 pg/ml; *n* = 27; *P* < 0.0001) (Figure 3B).

Moreover, no correlation was identified between plasma IL-22 and IL-6 levels with circulating Th22 frequency in newly-diagnosed B-NHL patients. Interestingly, plasma IL-22 was positively correlated with plasma TNF-α in newly-diagnosed B-NHL patients (*r* = 0.7422; *P* = 0.0015) (Figure 3C), while TNF-α level was unchangeable (*P* = 0.8996) (Figure 3D).

### The correlation of circulating Th22 cells with the clinical characteristics of patients with B-NHL

The relationship between circulating Th22 cells and the clinical characteristics across the study population were shown in Table 1. Here, B-NHL patients with older age (> 60 yr) exhibited profoundly increased frequency of circulating Th22 cells (1.713 ± 0.2380%, *n* = 10) compared to patients with younger age (≤ 60 yr) (1.039 ± 0.1402%, *n* = 16, *P* = 0.0152). However, there was no relationship between Th22 frequency and patient gender, serum lactate dehydrogenase (LDH) level or Ann Arbor staging classification.

**Table 1:** The correlations of Th22 frequency with clinical characteristics of B-NHL patients

Characteristics	*N*	Th22 (%)
Age/year*		
≤ 60 y	16	1.039 ± 0.1402
> 60 y	10	1.713 ± 0.2380
*P* value		0.0152
**Gender**		
Male	16	1.323 ± 0.1524
Female	10	1.258 ± 0.2790
*P* value		0.8255
**Serum LDH level**		
≤ Normal	14	1.265 ± 0.2210
> Normal	12	1.336±0.1671
*P* value		0.8039
**Ann Arbor staging**		
I + II	10	1.375 ± 0.2069
III + IV	16	1.250 ± 0.1899
*P* value		0.6717

The frequency of Th22 cells is presented as mean ± SEM.

LDH, lactate dehydrogenase;

* *P* < 0.05.

### The correlation of circulating Th22 cells with the development of B-NHL patients

**Figure 4:**
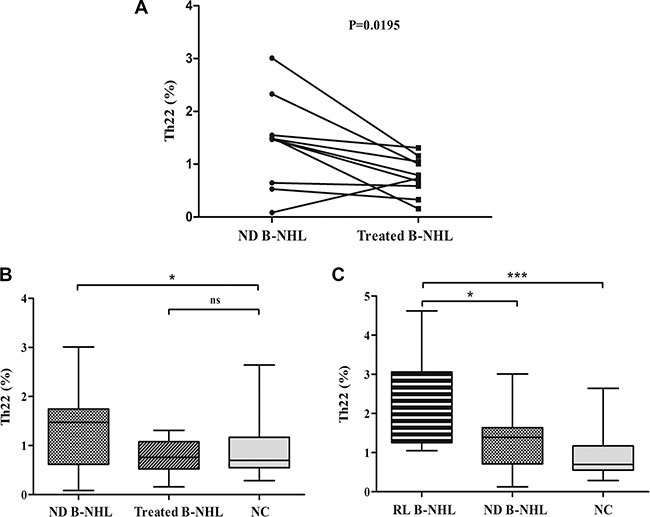
The frequency of circulating Th22 cells in patients after treatment (**A**) The frequency of Th22 cells in treated patients was significantly decreased compared with that before any chemotherapy. (**B**) Circulating Th22 frequency was normalized after treatment in B-NHL. (**C**) Circulating Th22 cells were dramatically elevated in relapsed patients (RL B-NHL) compared with newly-diagnosed B-NHL patients (ND B-NHL) and normal individuals (NC). *P < 0.05; ***P < 0.001; ns, non-significant.

We detected the frequency of circulating Th22 cells of 10 patients with B-NHL after one or two cycles of chemotherapeutic treatment. Th22 frequency in treated patients was significantly lower than that before any chemotherapy (*P* = 0.0195) (Figure 4A). And elevated circulating Th22 frequency was recovered to normal level after chemotherapeutic treatment (*P* = 0.5059) (Figure 4B). Meanwhile, we further analyzed the frequency of Th22 cells in other 9 relapsed B-NHL patients and found a remarkable increase in relapsed patients (1.81%; 1.05–4.62%; *n* = 9; *P* = 0.0434, *P* = 0.0004) compared with newly-diagnosed patients and normal controls (Figure 4C).

The typical dot plots of Th22 cells in representative treated and relapsed patients were shown in Figure 1G and 1H.

### The correlation of circulating Th22 cells with the therapy response of B-NHL patients

**Figure 5:**
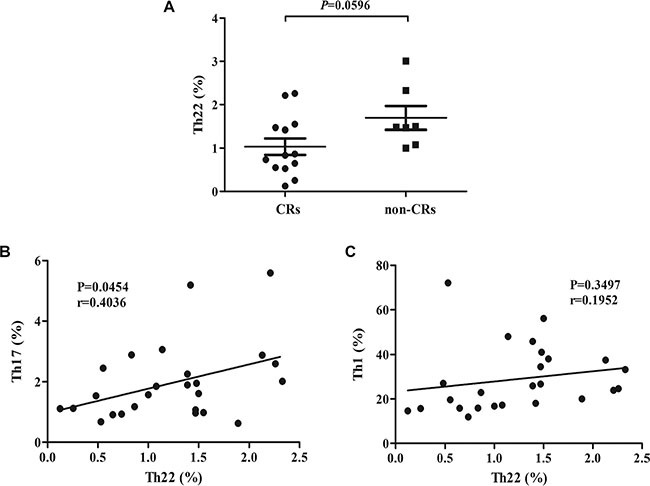
(**A**) A significant increase of Th22 frequency was found in newly-diagnosed B-NHL patients who achieved complete remission (CR) than others (non-CR). (**B**) A positive correlation was found between circulating Th22 and Th17 in B-NHL. (**C**) There was no correlation between circulating Th22 and Th1 in B-NHL.

For assessment of response, we used the International Workshop Response Criteria or its revised version [24, 25]. In our research, 20 of 26 newly-diagnosed B-NHL patients were treated with chemotherapy. After completed four cycles of chemotherapy, 13 patients (13/20, 65%) obtained a complete remission (CR), 4 patients a partial remission (PR) (4/20, 20%) and the remaining 3 patients (3/20, 15%) had a stable disease (SD). And we found that the frequency of circulating Th22 cells in newly-diagnosed B-NHL patients who achieved CRs was lower than that in the others (*P* = 0.0596) (Figure 5A). Furthermore, according to Th22 frequency in the total 26 newly-diagnosed B-NHL patients, low and high Th22 group was segregated by using median cut-off point. And the CR rate of low Th22 group (8/10, 80%) was higher than that of high Th22 group (5/10, 50%) after four cycles of chemotherapy.

### Th22 frequency showed a positive correlation with Th17 in newly-diagnosed B-NHL patients

Our previous study have showed that the frequency of circulating Th17 cells was lower in B-NHL patients compared with normal controls while circulating Th1 frequency was increased [26]. Interestingly, Th22 frequency showed a significantly positive correlation with Th17 in newly-diagnosed B-NHL patients (*r* = 0.4036; *P* = 0.0454) (Figure 5B). However, Th1 frequency failed to show a significant correlation with Th22 in B-NHL (*r* = 0.1952; *P* = 0.3497) (Figure 5C).

## DISCUSSION

Recently, understanding how the immune system influences tumor development and progression remains one of the most challenging questions in oncology.

Th22 cells are now defined as CD4^+^IFN-γ^−^IL-17^−^IL-22^+^ cells, which is a newly-identified and an independent Th subset compared with well-known Th1 and Th17 subset [5, 6, 27]. Many studies have reported that Th22 cells play important roles in tumors. In this study, we found the notably elevated frequency of Th22 cells in peripheral blood of B-NHL patients compared with healthy individuals. Moreover, Th22 frequency was significantly decreased and returned to normal level after one or two cycles of chemotherapy. Consistently, Wang et al. also detected that the increased Th22 cells in multiple myeloma (MM) patients were remarkably reduced after chemotherapy [28]. Our previous studies also revealed that aberrant Th22 cells population was recovered after treatment or complete remission in chronic and acute myeloid leukemia [29, 30]. Furthermore, another study focused on MM showed that the percentage of IL-22^+^IL-17^−^IL-13^+^ T cells was increased in relapsed/refractory patients compared with stage I/II patients [31]. Similarly, we found a significant increase of Th22 frequency in relapsed B-NHL patients compared with newly-diagnosed patients and normal controls. These findings collectively suggested that Th22 cells may participate in the development of B-NHL and measurement of Th22 frequency could be used in evaluating therapeutic effect.

Subsequently, our results also showed that Th22 frequency in older B-NHL patients (> 60 yr) was significantly higher than that in younger patients (≤ 60 yr). Although there are many clinically important prognostic factors in B-cell non-Hodgkin's lymphoma, advancing age was proven to be associated with shorter survival in many studies [32, 33]. In gastric and pancreatic cancer, increased intratumoral IL-22 producing CD4^+^ T cells correlated with disease's progression and predicted poor patient survival [22, 34]. Circulating Th22 cells were elevated and associated with lymph node metastases in cervical cancer [23]. Similarly, we found an increased frequency of Th22 cells correlated with a poorer response to chemotherapy in B-NHL patients. Above all, circulating Th22 frequency might be considered as a predicting factor for clinical outcome and prognosis of B-NHL patients.

In consistent with increased circulating Th22 frequency, significantly elevated plasma IL-22 concentration was observed in newly-diagnosed B-NHL patients, without correlating with Th22 frequency. IL-22 was produced by activated CD4^+^ T cells, CD8^+^ T cells, NKT cells, lymphoid tissue inducer and so on [19], implying that the plasma IL-22 level in B-NHL patients may not be determined by Th22 cells alone. Besides, plasma IL-22 was positively correlated with plasma TNF-α in B-NHL, accompanied with normal TNF-α level. Previous studies have demonstrated that Th22 cells can produce IL-22 and TNF-α [5, 6], however TNF-α is produced from various kinds of cells such as lymphoid cells, endothelial cells, mast cells and so on [35]. These may explain that plasma TNF-α level in B-NHL patients was comparable with that in healthy individuals. Additionally, a positive correlation (*r* = 0.5692; *P* = 0.0057) between plasma IL-22 and TNF-α was found in normal individuals (Supplementary Figure S1C). And the correlation in B-NHL patients was more relevant than that in normal individuals, indicating that the synergistic role between IL-22 and TNF-α may be more important in B-NHL.

In our current study, there existed a positive correlation between Th22 and Th17 cells in peripheral blood of newly-diagnosed B-NHL patients, implying that differentiation of Th22 and Th17 subset might be driven in an influential manner in B-NHL. This is consistent with several results found in rheumatoid arthritis, immune thrombocytopenia, cervical cancer and acute lymphoblastic leukemia [17, 23, 36, 37]. Furthermore, we observed a significantly decreased frequency of peripheral Th17 cells in B-NHL patients compared with healthy individuals and Th17 cells normalized after one or two cycles of chemotherapy [26]. Recently, several studies suggested that tumor microenvironments might selectively recruit Th17 cells and promote them trafficking from the periphery into tumor sites [4]. However, no previous study has shown data about this phenomenon in B-NHL. Thus, we hypothesized that tumor microenvironments in B-NHL might promote Th17 cells trafficking from the periphery into tumor sites. In order to investigate this hypothesis, the frequencies of intratumoral Th22 and Th17 cells in B-NHL should be measured in further research.

In conclusion, our study revealed elevated frequency of circulating Th22 cells in newly-diagnosed B-NHL patients for the first time. Moreover, Th22 cells were associated with B-NHL patients' ages and responses to chemotherapy. In addition, elevated Th22 cells were recovered after chemotherapy. Collectively, our data demonstrated that circulating Th22 frequency was associated with the clinical outcome and prognosis of B-NHL patients, indicating that Th22 immune response might be play an important role in the development and progression of B-NHL.

## MATERIALS AND METHODS

### Patients and controls

In this study, a total of 56 patients with lymphoma were collected from January 2013 to December 2013 at the Department of Hematology, Qilu Hospital, Jinan, China. All cases were consistent with lymphoma diagnostic criteria. The sources of patient-derived material and data are summarized in Tables 2 and 3. Thirty-nine healthy controls (25 females and 14 males, age range 20–67 years old, median 35 years old) were included. Ethical approval for the study was obtained from the Medical Ethical Committee of Qilu Hospital, Shandong University. Written informed consent was obtained from all participants.

**Table 2:** The classification information of patients with lymphoma in this study

Patients	Types	Subtypes	Numbers
Newly Diagnosed	B-NHL		26
		Diffuse large B-cell lymphoma	16
		Follicular cell lymphoma (I/II grade)	5
		Mantle cell lymphoma	1
		Chronic lymphocytic leukemia/small lymphocytic lymphoma	2
		Marginal zone B-cell lymphomas	2
	T-NHL		11
		Anaplastic large cell lymphoma	4
		Angioimmunoblastic T-cell lymphoma	7
	HL		10
		Nodular sclerosis classical HL	7
		Mixed cellularity classical HL	3
Relapsed	B-NHL		9
		Diffuse large B-cell lymphoma	6
		Follicular cell lymphoma	1
		Mantle cell lymphoma	2
Total			56

B-NHL, B-cell non-Hodgkin's lymphoma; T-NHL, T-cell non-Hodgkin's lymphoma; HL, Hodgkin's lymphoma.

**Table 3:** Clinical characteristics of newly-diagnosed patients with lymphoma in this study

Variable	B-NHL	T-NHL	HL
Age, yr	52 (12)	53 (17)	27.5 (18.2)
Gender			
Male	16 (61.5)	7 (63.6)	7 (70)
Female	10 (38.5)	4 (36.4)	3 (30)
Hemoglobin, g/L			
≥ 120	19 (73.1)	6 (54.5)	7 (70)
< 120	7 (26.9)	5 (45.5)	3 (30)
ESR, mm/h			
Normal	11 (42.3)	2 (18.2)	3 (30)
> Normal	10 (38.5)	7 (63.6)	5 (50)
Unknown	5 (19.2)	2 (18.2)	2 (20)
Serum LDH, U/L			
Normal	14 (53.8)	5 (45.5)	7 (70)
> Normal	12 (46.2)	6 (54.5)	3 (30)
Ann Arbor staging			
I + II	10 (38.5)	4 (36.4)	4 (40)
III + IV	16 (61.5)	7 (63.6)	6 (60)
Bone marrow involvement			
(–)	21 (80.8)	10 (91.9)	9 (90)
(+)	5 (19.2)	1 (9.1)	1 (10)
B symptoms			
(–)	20 (76.9)	4 (36.4)	6 (60)
(+)	6 (23.1)	7 (63.6)	4 (40)

Ages are presented as median (SD) and the other characteristics are presented as *n* (%). B-NHL, B-cell non-Hodgkin's lymphoma; T-NHL, T-cell non-Hodgkin's lymphoma; HL, Hodgkin's lymphoma.

### Treatment regimens and samples collection

All patients with lymphoma were treated with a standard induction chemotherapy based on newly NCCN guideline. All treated patients and relapsed patients that were mentioned in our study were treated with CHOP (cyclophosphamide, doxorubicin, vincristine, prednisone) regimen with or without rituximab. In order to avoid the drugs influence on the distribution of Th subset, we collected peripheral blood samples from newly diagnosed patients before any treatment. And the collection of blood samples from treated and relapsed patients took place after at least two weeks when they received chemotherapy.

### Flow cytometric analysis

Flow cytometry was used to study membrane makers and intracellular cytokines to identify the cytokine-producing cells. Briefly, heparinized peripheral blood with an equal volume of 1640 medium (Hyclone, USA) was incubated for 4 h at 37°C, 5% CO2 in the presence of 25 ng/ml of phorbol myristate acetate (PMA), 1 μg/ml of ionomycin, and 1.7 μg/ml monensin (all from Alexis Biochemicals, USA). After incubation, the cells were stained with Alexa Fluor^®^ 647 or PerCP/Cy 5.5 anti-CD4 monoclonal antibody at room temperature in the dark for 20 min. After surface staining, the cells were next stained with FITC anti-IFN-γ, PerCP/Cy 5.5 or PE anti-IL-17 and PE or Alexa Fluor^®^ 660 anti-IL-22 monoclonal antibodies after fixation and permeabilization. All the antibodies were purchased from Biolegend and eBioscience (California, USA). Fixation and permeabilizaton reagents were purchased from eBioscience (California, USA). Isotype controls were given to enable correct compensation and confirm antibody specificity. Stained cells were analyzed by flow cytometric analysis using a FACS Calibur cytometer equipped with CellQuest software (BD Bioscience PharMingen, USA). For analysis, we first gated lymphocytes, then gated CD4^+^IFN-γ^−^ T cells in lymphocytes, and analyzed the percentages of CD4^+^IFN-γ^−^IL-17^−^IL-22^+^ (Th22) cells in CD4^+^IFN-γ^−^ T cells.

### Enzyme-Linked Immunosorbent Assay (ELISA)

Peripheral blood was collected into heparin-anticoagulant vacutainer tubes. All plasma specimens were obtained from all subjects by centrifugation and stored at −80°C for determination of cytokines. The concentrations of IL-22, IL-6 and TNF-α in each group were determined with a quantitative sandwich enzyme immunoassay technique in accordance with the manufacturer's recommendations (eBioscience, USA). The concentrations were calculated from a standard curve according to the manufacturer's protocol. Sensitive concentration of IL-22, IL-6 and TNF-α ELISA kit is 5 pg/ml, 0.92 pg/ml and 2.3 pg/ml respectively.

### Statistical analysis

The results were expressed as median range. Student *t*-test, Wilcoxon signed rank test, Mann-Whitney *U* test or Kruskal-Wallis test was applied to determine significant differences between groups. All tests were performed by GraphPad Prism 5.0 system. In these analyses, two-sided *P*-values < 0.05 were considered to be statistically significant.

## SUPPLEMENTARY MATERIALS FIGURE



## References

[1] Zhang Y, Ma D, Zhang Y, Tian Y, Wang X, Qiao Y, Cui B (2011). The imbalance of Th17/Treg in patients with uterine cervical cancer. Clin Chim Acta.

[2] Goedegebuure PS, Eberlein TJ (1995). The role of CD4+ tumor-infiltrating lymphocytes in human solid tumors. Immunol Res.

[3] Saxena R, Kaur J (2015). Th1/Th2 cytokines and their genotypes as predictors of hepatitis B virus related hepatocellular carcinoma. World J Hepatol.

[4] Bailey SR, Nelson MH, Himes RA, Li Z, Mehrotra S, Paulos CM (2014). Th17 cells in cancer: the ultimate identity crisis. Front Immunol.

[5] Trifari S, Kaplan CD, Tran EH, Crellin NK, Spits H (2009). Identification of a human helper T cell population that has abundant production of interleukin 22 and is distinct from T(H)-17, T(H)1 and T(H)2 cells. Nat Immunol.

[6] Duhen T, Geiger R, Jarrossay D, Lanzavecchia A, Sallusto F (2009). Production of interleukin 22 but not interleukin 17 by a subset of human skin-homing memory T cells. Nat Immunol.

[7] Dumoutier L, Louahed J, Renauld JC (2000). Cloning and characterization of IL-10-related T cell-derived inducible factor (IL-TIF), a novel cytokine structurally related to IL-10 and inducible by IL-9. J Immunol.

[8] Chung Y, Yang X, Chang SH, Ma L, Tian Q, Dong C (2006). Expression and regulation of IL-22 in the IL-17-producing CD4+ T lymphocytes. Cell Res.

[9] Xie MH, Aggarwal S, Ho WH, Foster J, Zhang Z, Stinson J, Wood WI, Goddard AD, Gurney AL (2000). Interleukin (IL)-22, a novel human cytokine that signals through the interferon receptor-related proteins CRF2-4 and IL-22R. J Biol Chem.

[10] Bleicher L, de Moura PR, Watanabe L, Colau D, Dumoutier L, Renauld JC, Polikarpov I (2008). Crystal structure of the IL-22/IL-22R1 complex and its implications for the IL-22 signaling mechanism. FEBS Lett.

[11] de Oliveira Neto M, Ferreira JR, Colau D, Fischer H, Nascimento AS, Craievich AF, Dumoutier L, Renauld JC, Polikarpov I (2008). Interleukin-22 forms dimers that are recognized by two interleukin-22R1 receptor chains. Biophys J.

[12] Ziesche E, Bachmann M, Kleinert H, Pfeilschifter J, Muhl H (2007). The interleukin-22/STAT3 pathway potentiates expression of inducible nitric-oxide synthase in human colon carcinoma cells. J Biol Chem.

[13] Pickert G, Neufert C, Leppkes M, Zheng Y, Wittkopf N, Warntjen M, Lehr HA, Hirth S, Weigmann B, Wirtz S, Ouyang W, Neurath MF, Becker C (2009). STAT3 links IL-22 signaling in intestinal epithelial cells to mucosal wound healing. J Exp Med.

[14] Weber GF, Gaertner FC, Erl W, Janssen KP, Blechert B, Holzmann B, Weighardt H, Essler M (2006). IL-22-mediated tumor growth reduction correlates with inhibition of ERK1/2 and AKT phosphorylation and induction of cell cycle arrest in the G2-M phase. J Immunol.

[15] Kagami S, Rizzo HL, Lee JJ, Koguchi Y, Blauvelt A (2010). Circulating Th17, Th22, and Th1 cells are increased in psoriasis. J Invest Dermatol.

[16] Peng D, Xu B, Wang Y, Guo H, Jiang Y (2013). A high frequency of circulating th22 and th17 cells in patients with new onset graves' disease. PLoS One.

[17] Zhang L, Li JM, Liu XG, Ma DX, Hu NW, Li YG, Li W, Hu Y, Yu S, Qu X, Yang MX, Feng AL, Wang GH (2011). Elevated Th22 cells correlated with Th17 cells in patients with rheumatoid arthritis. J Clin Immunol.

[18] Azizi G, Yazdani R, Mirshafiey A (2015). Th22 cells in autoimmunity: a review of current knowledge. Eur Ann Allergy Clin Immunol.

[19] Jia L, Wu C (2014). The biology and functions of Th22 cells. Adv Exp Med Biol.

[20] Qin S, Ma S, Huang X, Lu D, Zhou Y, Jiang H (2014). Th22 cells are associated with hepatocellular carcinoma development and progression. Chin J Cancer Res.

[21] Liu T, Peng L, Yu P, Zhao Y, Shi Y, Mao X, Chen W, Cheng P, Wang T, Chen N, Zhang J, Liu X, Li N (2012). Increased circulating Th22 and Th17 cells are associated with tumor progression and patient survival in human gastric cancer. J Clin Immunol.

[22] Zhuang Y, Peng LS, Zhao YL, Shi Y, Mao XH, Guo G, Chen W, Liu XF, Zhang JY, Liu T, Luo P, Yu PW, Zou QM (2012). Increased intratumoral IL-22-producing CD4(+) T cells and Th22 cells correlate with gastric cancer progression and predict poor patient survival. Cancer Immunol Immunother.

[23] Zhang W, Tian X, Mumtahana F, Jiao J, Zhang T, Croce KD, Ma D, Kong B, Cui B (2015). The existence of Th22, pure Th17 and Th1 cells in CIN and Cervical Cancer along with their frequency variation in different stages of cervical cancer. BMC Cancer.

[24] Cheson BD, Horning SJ, Coiffier B, Shipp MA, Fisher RI, Connors JM, Lister TA, Vose J, Grillo-Lopez A, Hagenbeek A, Cabanillas F, Klippensten D, Hiddemann W (1999). Report of an international workshop to standardize response criteria for non-Hodgkin's lymphomas. NCI Sponsored International Working Group. J Clin Oncol.

[25] Cheson BD, Pfistner B, Juweid ME, Gascoyne RD, Specht L, Horning SJ, Coiffier B, Fisher RI, Hagenbeek A, Zucca E, Rosen ST, Stroobants S, Lister TA (2007). Revised response criteria for malignant lymphoma. J Clin Oncol.

[26] Lu T, Yu S, Liu Y, Yin C, Ye J, Liu Z, Ma D, Ji C (2016). Aberrant Circulating Th17 Cells in Patients with B-Cell Non-Hodgkin's Lymphoma. PLoS One.

[27] Eyerich S, Eyerich K, Pennino D, Carbone T, Nasorri F, Pallotta S, Cianfarani F, Odorisio T, Traidl-Hoffmann C, Behrendt H, Durham SR, Schmidt-Weber CB, Cavani A (2009). Th22 cells represent a distinct human T cell subset involved in epidermal immunity and remodeling. J Clin Invest.

[28] Wang M, Chen P, Jia Y, He N, Li D, Ji C, Ma D (2015). Elevated Th22 as well as Th17 cells associated with therapeutic outcome and clinical stage are potential targets in patients with multiple myeloma. Oncotarget.

[29] Chen P, Wang M, Li D, Jia Y, He N, Li W, Ma D, Ji C (2015). The alteration and clinical significance of Th22/Th17/Th1 cells in patients with chronic myeloid leukemia. J Immunol Res.

[30] Yu S, Liu C, Zhang L, Shan B, Tian T, Hu Y, Shao L, Sun Y, Ji C, Ma D (2014). Elevated Th22 cells correlated with Th17 cells in peripheral blood of patients with acute myeloid leukemia. Int J Mol Sci.

[31] Di Lullo G, Marcatti M, Heltai S, Brunetto E, Tresoldi C, Bondanza A, Bonini C, Ponzoni M, Tonon G, Ciceri F, Bordignon C, Protti MP (2015). Th22 cells increase in poor prognosis multiple myeloma and promote tumor cell growth and survival. Oncoimmunology.

[32] Moore DF, Cabanillas F (1998). Overview of prognostic factors in non-Hodgkin's lymphoma. Oncology (Williston Park).

[33] Minard-Colin V, Brugieres L, Reiter A, Cairo MS, Gross TG, Woessmann W, Burkhardt B, Sandlund JT, Williams D, Pillon M, Horibe K, Auperin A, Le Deley MC (2015). Non-Hodgkin Lymphoma in Children and Adolescents: Progress Through Effective Collaboration, Current Knowledge, and Challenges Ahead. J Clin Oncol.

[34] Xu X, Tang Y, Guo S, Zhang Y, Tian Y, Ni B, Wang H (2014). Increased intratumoral interleukin 22 levels and frequencies of interleukin 22-producing CD4+ T cells correlate with pancreatic cancer progression. Pancreas.

[35] Kapadia S, Lee J, Torre-Amione G, Birdsall HH, Ma TS, Mann DL (1995). Tumor necrosis factor-alpha gene and protein expression in adult feline myocardium after endotoxin administration. J Clin Invest.

[36] Hu Y, Li H, Zhang L, Shan B, Xu X, Li H, Liu X, Xu S, Yu S, Ma D, Peng J, Hou M (2012). Elevated profiles of Th22 cells and correlations with Th17 cells in patients with immune thrombocytopenia. Hum Immunol.

[37] Tian T, Sun Y, Li M, He N, Yuan C, Yu S, Wang M, Ji C, Ma D (2013). Increased Th22 cells as well as Th17 cells in patients with adult T-cell acute lymphoblastic leukemia. Clin Chim Acta.

