# Supplementary material for: Increased frequency of circulating Th22 cells in patients with B-cell non-Hodgkin's lymphoma
Source: Oncotarget. 2016 Jul 30;7(35):56574–83. doi: 10.18632/oncotarget.10966 (PMC5302935; doi:10.18632/oncotarget.10966)
Supplement: Supplementary file 1 [file oncotarget-07-56574-s001.pdf]

Increased frequency of circulating Th22 cells in patients with B-cell non-Hodgkin's lymphoma

Supplementary Materials

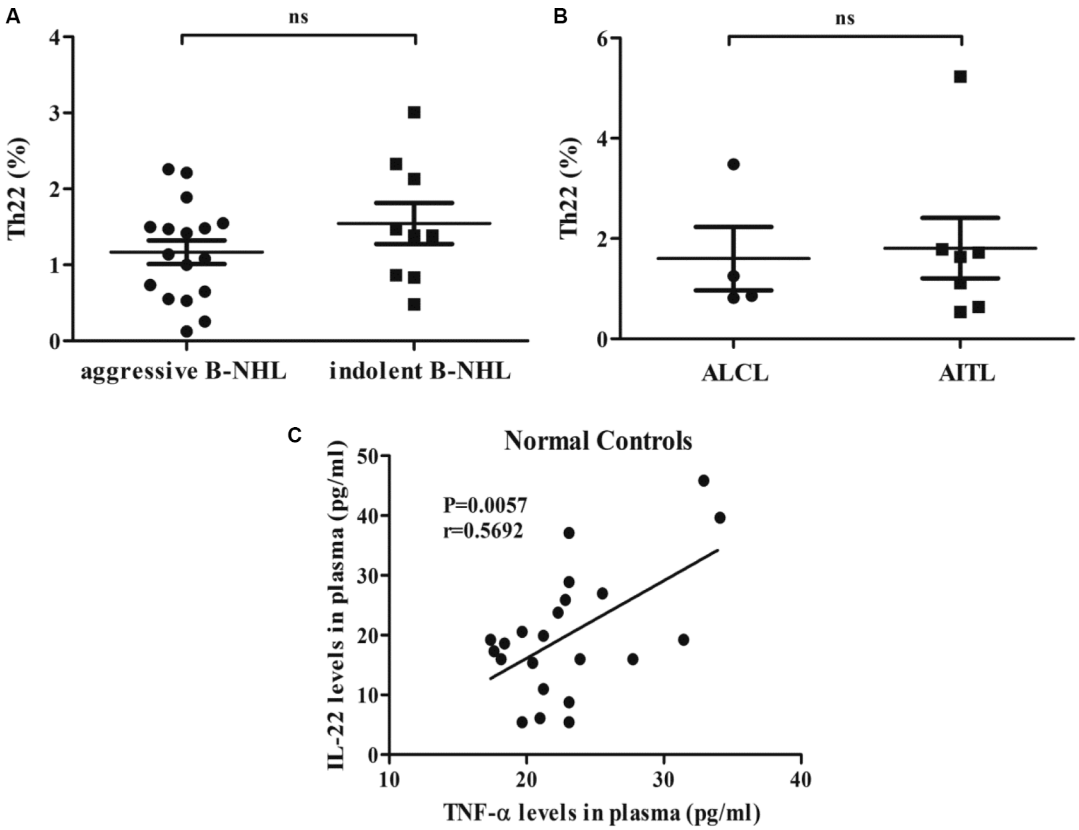

Supplementary Figure S1: (**A**) There was no significant difference of Th22 frequency between aggressive and indolent B-NHL. (**B**) There was no significant difference of Th22 frequency between anaplastic large cell lymphoma (ALCL) and angioimmunoblastic T-cell lymphoma (AITL). (**C**) A positive correlation was found between plasma IL-22 and TNF-α in normal controls.
